# Can Community Members Identify Tropical Tree Species for REDD+ Carbon and Biodiversity Measurements?

**DOI:** 10.1371/journal.pone.0152061

**Published:** 2016-11-04

**Authors:** Mingxu Zhao, Søren Brofeldt, Qiaohong Li, Jianchu Xu, Finn Danielsen, Simon Bjarke Lægaard Læssøe, Michael Køie Poulsen, Anna Gottlieb, James Franklin Maxwell, Ida Theilade

**Affiliations:** 1 Key Laboratory for Plant Diversity and Biogeography of East Asia, Kunming Institute of Botany, Chinese Academy of Sciences, Kunming, Yunnan, China; 2 University of Chinese Academy of Sciences, Beijing, China; 3 World Agroforestry Centre, East and Central Asia, Kunming, Yunnan, China; 4 Department of Food and Resource Economics, Faculty of Science, University of Copenhagen, Copenhagen, Denmark; 5 Nordic Foundation for Development and Ecology (NORDECO), Copenhagen, Denmark; 6 Biology Department, Faculty of Science, Chiang Mai University, Chiang Mai, Thailand; CIFOR Headquarters, INDONESIA

## Abstract

Biodiversity conservation is a required co-benefit of REDD+. Biodiversity monitoring is therefore needed, yet in most areas it will be constrained by limitations in the available human professional and financial resources. REDD+ programs that use forest plots for biomass monitoring may be able to take advantage of the same data for detecting changes in the tree diversity, using the richness and abundance of canopy trees as a proxy for biodiversity. If local community members are already assessing the above-ground biomass in a representative network of forest vegetation plots, it may require minimal further effort to collect data on the diversity of trees. We compare community members and trained scientists’ data on tree diversity in permanent vegetation plots in montane forest in Yunnan, China. We show that local community members here can collect tree diversity data of comparable quality to trained botanists, at one third the cost. Without access to herbaria, identification guides or the Internet, community members could provide the ethno-taxonomical names for 95% of 1071 trees in 60 vegetation plots. Moreover, we show that the community-led survey spent 89% of the expenses at village level as opposed to 23% of funds in the monitoring by botanists. In participatory REDD+ programs in areas where community members demonstrate great knowledge of forest trees, community-based collection of tree diversity data can be a cost-effective approach for obtaining tree diversity information.

## Introduction

Biologists working in the tropics and elsewhere have always relied on local people for guidance. Indigenous and local communities possess knowledge about the landscape they inhabit [1]. In tropical forests, indigenous cultures sometimes have meticulous classification systems to distinguish between vegetation types on the landscape [2–8].

While individual human societies may differ considerably in their conceptualization of plants and animals, folk biological classification systems share a number of strikingly similar structural principles [9,10]. All languages seem to have linguistically recognised groupings of organisms (or taxa) of varying degree of inclusiveness, and all languages seem to group the taxa into hierarchical categories. Moreover, taxa assigned to each rank are usually mutually exclusive, and folk biology taxa are about as inclusive as the scientific genera [11].

Few studies have investigated the salience of folk classification of vegetation and plants. From our review, field data on plant species collected by community members and those collected by botanists were compared in only five studies (summary in Table 1). While scientific plant names are designed to prevent the same name from being used for different species, such rules do not apply to vernacular names [12]. Local people often split taxa of great cultural significance into many ethnoforms while species that are less important or less distinctive are often lumped into one single ethnoform with a common name [13,14]. Local names are often based on different criteria from those of scientific taxonomy, such as use or spiritual status [12]. Vernacular names cannot thus be equated consistently to particular scientific names [12]. Nevertheless, some vernacular names do show a one-to-one correspondence with scientific taxa. For example, Jinxiu et al. [15] found a high correlation between folk and scientific plant species among the Dai people of Xishuangbanna, China (Table 1). Likewise, Cardoso et al. [16] found the classification criteria used for fungi by several Brazilian indigenous groups to be similar to those used in classical, morphology-based scientific studies.

**Table 1:** Previous scientific studies comparing community members’ classification of vegetation and identification of plant species to those of scientists.

Scale	Authors	Vegetation type and country	Villages, ethnic groups (number) or area (ha)	Methods	Characteristics of community monitors	Statistics employed*	Attribute	Result of comparison between local community member and trained scientist surveys
Vegetation type	Hellier et al. 1999 [58]]	Pine—oak forest.Mexico	2 villages, 2 ethnic groups	Interviews	57 persons incl. 10 women and 47 men	No	Forest cover and harvested species	Some contradiction on vegetation change
Vegetation type	Naidoo & Hill 2006 [59]	Atlantic forest. Paraguay	64.000 ha	Field survey and satellite images	Some community members were employed as park rangers. No numbers were available	Yes	Vegetation classes	Vegetation classified by community members similar to scientist-led, locally supervised classification of satellite images
Vegetation type	Chalmers & Fabricius 2007 [60]	Grassland, woodland, and forest. South Africa	1660 ha	Interviews	51 persons. 11 were recognised as local experts by the local community; 40 were randomly selected	No	Forest and woodland cover change	Local ‘experts’ assessment corresponded with scientists. Randomly selected community members had shallower knowledge
Vegetation type	Halme & Bodmer 2007 [61]	Tropical rainforest. Peru	1 village	Interviews	26 shifting cultivators, fishers and hunters. Used to collaborate w. scientists	Yes	Forest types	Close correspondence between forest type classification by communities and floristic classification by botanists (Pteridophytes used as indicator taxon)
Vegetation type	Vergara-Asenjo et al. 2015 [62]	Tropical rainforest. Panama	3 villages	Workshop and interviews	95 indigenous technicians trained in forest mensuration	Yes	Ten land-cover classes	Digital processing of RapidEye imagery compared to participatory land-cover map. In forested areas, accuracy of participatory classification was significantly better than classification based on digital image processing
Plant species	Wilkie & Saridan 1999 [12]	Tropical rainforest. Indonesia	1 ethnic group.1 ha	Field survey	2 shifting cultivators, 46 and 66 years old men. One had worked in a logging company	No	Species identification Trees ≥10 cm dbh	Vernacular names could not be equated consistently to taxa identified by scientists
Plant species	Jinxiu et al 2004 [15]	Tropical rainforest. China	1 ethnic group. 1600 ha	Field survey	6 Dai villagers, about 40 years old	No	Plant species identification	High correspondence between folk and scientific plant species identification
Plant species	Lacerda et al. 2010 [14]	Tropical rainforest. Brazil	1 ethnic group. 546 ha	Field survey	NA	No	Species identification Trees >45 cm dbh	Local people’s identifications matched those of scientists. Conversely, matching of vernacular names to scientific names using a pre-existing, non-specific list, used by timber companies was severely deficient
Plant species	Oldekop et al. 2011 [63]	Tropical rainforest. Ecuador	2 ethnic groups. 0.05 ha	Field survey	20 persons, 18–55 years old, from 9 indigenous and settler communities. All had received visual guides or hands-on training	Yes	Species richness of ferns	Strong correlation of species richness estimates between the community members and the scientists
Plant species	Theilade et al. 2015 [64]	Tropical rainforest. Indonesia	1 ethnic group	Field survey	11 Dayak men, 20–30 years old; 6 of them had worked in logging companies	Yes	Species identification. Trees >10 cm dbh	Vernacular names could not be equated consistently to taxa identified by scientists

*) Statistics used to compare community member and scientist-executed classification or identification.

NA = No information available.

Deforestation and forest degradation in the tropics are responsible for approximately 20% of anthropogenic carbon emissions [17] and compromise both livelihoods and biodiversity. In response, the United Nations Framework Convention on Climate Change (UNFCCC) has agreed to establish an international framework that will provide developing countries with financial incentives to reduce emissions from deforestation and forest degradation. While the primary purpose of this framework, known as REDD+ (Reducing Emissions from Deforestation and Forest Degradation), is climate change mitigation, it may have enormous co-benefits for biodiversity because tropical forests are exceptionally rich in exclusive biodiversity reservoirs [18]. However, REDD+ may have a negative influence on biodiversity when low-carbon, high-biodiversity forests are replaced with high-carbon, low-biodiversity forests (e.g. tree plantations), or when the protection of high-carbon forest in one area leads to the displacement of other high-biodiversity forests [19, 20]. Biodiversity monitoring is therefore needed [21–23].

A key obstacle in accounting for biodiversity is a lack of consensus as to what to monitor [24] because there is, so far, no single, agreed metric of biodiversity, unlike carbon (Mg ha^-1^ of carbon). While biomass estimates are often based on number of trees per hectare and their diameter at breast height (DBH), monitoring of biodiversity requires understanding parameters such as species richness, composition, abundance and the distribution of many taxa, which is a tall order given the widespread lack of human and financial resources [25]. One approach to minimizing the risk of overburdening REDD+ programs is to deploy a limited set of ‘indicator’ taxa [26]. The use of indicator taxa in biodiversity monitoring for REDD+ needs to satisfy the following four requirements: low monitoring costs, ease of identification, surrogates of ecosystem integrity, and cross-taxon congruency, see below [26].

Tree assemblage, i.e. a community of canopy-tree species including species richness and abundance as attributes, has been suggested as a suitable indicator. Firstly, the sampling of trees is relatively easy and inexpensive [27]. REDD+ remote sensing relies on tree density and DBH to ground-truth satellite images. Tree density data will thus be collected irrespective of how biodiversity safeguards are monitored. Secondly, unlike most other organisms, tree taxonomy is relatively well described. Thirdly, tree assemblages have a high cross-taxon congruency, in which tree species richness and composition are correlated with those of other taxa [28, 29], probably because trees provide other taxa with resources and habitats.

Imai et al. [25] state that, for trees, the availability of local experts is relatively adequate compared with other taxa. While this may be true for some parts of the world, the enormous challenge of flora projects in lower-income countries, where the most diverse terrestrial ecosystems are found [30], is exacerbated by the short supply of taxonomic experts available [31–33]. Researchers have pointed to the vast number of indigenous and local botanical experts, representing a potentially valuable, yet largely unrecognised and untapped, resource [33–36]. The participation of ‘parataxonomists’, defined as resident, field-based, biodiversity inventory specialists with no formal training [37], has been shown to enhance biodiversity inventories for both arthropods [35,37–39], fungi [16,40], and plants [15,41,42].

UNFCCC texts and guidance documents on the technical aspects of REDD+ outline explicit roles for indigenous people and local communities in implementing REDD+ [[43–46]. Yet little has been published on how community-based REDD+ should be implemented in practice, including community-level monitoring of carbon, livelihoods or biodiversity [47,48]. The degree of local participation may vary from virtually no local involvement to an entirely local effort, with data collection, interpretation and reporting undertaken by local people [49]. Studies suggest that locally-based monitoring may be advantageous in terms of lower costs [50], enhanced local ownership, greater cultural relevance and improved institutional strength at the community level [51–53]. Moreover, local people’s participation in monitoring can potentially enhance decision-making at the operational level of forest management [54,55]. Community monitoring of forest carbon and tree biodiversity may therefore contribute to a fair and equitable REDD+ [47].

One of the functions of the newly-established Intergovernmental Science-Policy Platform on Biodiversity and Ecosystem Services (IPBES) is to bring different knowledge systems, including indigenous and local knowledge systems, to the science–policy interface [56]. One key challenge lies in how to use information generated by different knowledge systems within synthetic assessments at the science-policy interface [57]. It is therefore important to understand how folk biological classification systems connect or otherwise with scientific classification systems.

Here, we explore one aspect of folk biological classification systems of particular relevance to REDD+: we compare local community-collected data on canopy trees in forest vegetation plots with that collected by trained botanists, using the botanists’ findings as a benchmark. In addition, we compare the costs of trained botanists’ and community monitors’ identification of tree species.

## Methods

### Ethics Statement

This research did not involve human or other animal subjects. For plant collections, we collected the minimum number of specimens required to appropriately voucher field identifications. The field studies did not involve endangered or protected species.

Permission to conduct research in Manlin was obtained through a bilateral agreement between Kunming Institute of Botany (Chinese Academy of Sciences) and the Forestry Bureau of Xishuangbanna Autonomous Prefecture at the regular meeting between these parties. Free, prior and informed consent was obtained for all community monitors participating in the study.

### Literature Search

We reviewed previous studies that have compared scientist and local knowledge on vegetation or species level by searching the databases Web of Science, PubMed, CABI, AGRICOLA and AGRIS using the following keywords: participatory monitoring, local monitoring, community monitoring, parataxonomist, local ecological knowledge, traditional ecological knowledge, and indigenous knowledge.

### Study Site and Data Collectors

The study site was chosen opportunistically. The criteria were that the site was appointed by the government as a potential REDD+ site, and that local communities used the forest area. The study area was the forest near Man Lin village, located at 1180 m.a.s.l. (above sea level) in Xiangming township of Xishuangbanna autonomous prefecture, Yunnan province. The climate is monsoonal with an average annual temperature of 25° C and an average annual precipitation of 1700 mm. Slope inclinations range between 30° and 70°, and in some areas attain up to 90°. The vegetation is tropical mountain rainforest at around 900–1400 m.a.s.l. The forest area surveyed covered 761 ha and is characterised by *Castanopsis mekongensis* and *Schima wallichii*. The canopy can be divided into 3 layers: the overstory reaches 35 m in height and is dominated by emergent trees such as *Pometia pinnata;* the midstory reaches 25 m and is dominated by *Castanopsis* spp., and *Schima wallichii* while the understory contains a multitude of species, such as *Cratoxylon cochinchinensis*, *Phoebe puwenensis*, *Machilus* spp., *Lithocarpus* spp. *Elaeocarpus* spp. *Mallotus* spp. Shrub and herbaceous layers at the edges and inside forest areas are rich in species. Community members and scientists both measured two forest strata (homogenous forest areas in terms of structure and composition). The stratum closer to the village (291 ha) is classified as collective forest. It is moderately disturbed forest and consists of abandoned shifting cultivation fields and ancient tea trees with an overstory of natural forest. The second stratum (470 ha) is classified as state forest and consists mainly of natural old-growth forest on steep to very steep slopes. Few trees were being extracted 40–60 years ago. Shifting cultivation was practised in small plots on more gradual slopes from the 1950s to the1990s and then gradually abandoned. The forest recovered and is in a good condition with a profusion of lianas and epiphytes. Tea seedlings are planted in the collective forest and dominate the understory in smaller areas. Firewood to dry the tea leaves as well as timber for house construction and furniture are harvested from nearby private forests. The rural Yi community is connected by road and most villagers are employed in newly-established rubber plantations.

Data were collected from permanent vegetation plots. Plots were surveyed by botanists in July 2012 and community members in March 2013. Representatives of the local Yi community selected three community participants for tree species identification, based on their interest in and experience of forest resources. These community monitors are thus probably more skilled than the average villager. All community monitors were male, reflecting the fact that men visit the forest more frequently than women, often for hunting and collecting of non-timber forest products (NTFPs). Women do not venture into steeply-sloped forest areas distant from the village. All community monitors had attended primary school, which is the usual length of schooling in the village. The community monitors received 1–2 days’ training from an intermediate organisation (research institution) on how to establish plots and measure tree girth, as required for assessing the above-ground biomass. The community identification of trees relied solely on existing local ecological knowledge. The botanical team consisted of the late J.F. Maxwell, botanist and curator at Chiang Mai Herbarium (CMU), who had more than 40 years’ experience of floristic work in Indochina, and PhD fellow Mingxu Zhao from Kunming Institute of Botany (KIB).

### Methods for Measuring Forest Tree Diversity and Costs

The community monitors and the staff of the intermediate organisation divided the forest into two homogenous strata in terms of tree species composition and level of degradation, using the available knowledge of the forest and its history (i.e. previous logging or shifting cultivation). Based on this pre-analysis, staff of the intermediate organisation randomly placed 30 circular plots in each stratum making a total of 60 plots. The community monitors and professional botanists then independently carried out forest inventories in each plot. All trees with a girth of ≥ 30 cm (as a proxy for DBH ≥ 10 cm) were identified within a radius of 9 m from the plot centre and all trees with a girth of ≥ 100 cm (proxy for DBH ≥ 30 cm) were identified within a radius of 15 m from the plot centre. Local names and scientific names were recorded by pencil on pre-printed paper forms. The community monitors worked as a team and discussed identifications internally but not with the botanists. Botanists were allowed to ask local guides about flower and fruit characteristics and phenology as some trees were not in flower/fruit at the time of the survey. Specimens were collected for herbaria work by the botanists. Botanists used Flora of Thailand [65], Flora of Yunnan [66], and Flora of China [67] plus herbarium material at Xishuangbanna Botanic Garden (XTBG), Kunming Institute of Botany (KIB) and Chiang Mai (CMU). Voucher specimens were deposited at both CMU and KIB.

We estimated the costs of community-based and professionally-executed identifications on the basis of the actual expenses incurred for local transport and during the training and fieldwork [47,50]. The cost of tree species identification was accounted for separately from other research activities. The chief botanist’s airfare from Thailand to China was not included. We calculated the number of genera and species identified by both community monitors and botanists.

## Results

### Tree Identification by Botanists and Community Monitors

In total, 1071 trees were recorded by both the botanists and the community monitors (S1 File). We first examined how many taxa and morphospecies (species distinguished from others only by their morphology) the botanists could identify. We found that, of the 1071 trees, the botanists were able to identify 1052 trees belonging to 50 families, 104 genera, and 142 species. In addition, the botanists recognised 19 morphospecies (1 identified to family level, 7 to genus level and 11 unidentified). Of the 161 recognised taxa, the botanists named 149 to genus level and 142 to species level.

We found no significant difference between the number of trees in the plot network identified to at least genus level by botanists (99.3%; n = 1071 trees) and community monitors (94.7%; *n* = 1071 trees). The community monitors were able to name 1013 trees belonging to 42 families, 90 genera, and 111 species that showed a one-to-one correspondence with the botanists’ named genera and species. Of the 161 taxa recognised by the botanists, the community monitors named 128 to genus level and 111 to species level (Table 2).

**Table 2:** Comparison of the number of trees identified to genus or species level by botanists and by community monitors, and the number of genera and species that the identified trees belonged to, in montane forest in Yunnan, China (*n* = 1071 trees). Numbers for community monitors are calculated using only those with a one-to-one correspondence to scientific taxa.

Number identified	Botanists	Community monitors
Trees to genus level	1052	1013
Trees to species level	1037	800
Genera	149	128
Species	142	111

Community monitors grouped 27 species (262 trees), mainly of the genera *Castanopsis*, *Engelhardtia*, *and Schima*, into 11 ethnotaxa. The 11 ethnotaxa referred one-to-one to 11 scientific genera. The lumping of species that morphologically appear very similar makes up half (52%, *n* = 31) the difference between the number of species identified by botanists and community monitors. Community monitors split 2 species (7 trees) into four ethnospecies. In addition, the community monitors did not have a name for 58 trees (5%, *n* = 1071 trees) belonging to 32 species. We found that 3 trees (0.3%, *n* = 1071 trees) seemed to be misidentified by the community monitors based on the observation that the community monitors consistently identified other trees of the same species as being of a different ethnospecies.

We investigated whether the trees that the community monitors did not have a name for shared any common characteristics that might make them difficult or irrelevant for them to identify. We examined the composition of the unnamed trees against six criteria: family, wood density, size, habitat (primary and secondary forest), abundance, and usefulness for the community members as source of timber, fruits and other products.

The 58 unidentified trees belonged to what the botanists identified as 25 genera of 18 families. Five families represented 57% of the unidentified trees (*Magnoliaceae*, *Meliaceae*, *Myristicaceae*, *Rubiaceae*, and *Rutaceae*). Forty-five (78%, *n* = 58) of the trees were rare, i.e. only 1–3 individuals were encountered in the plot network. Thirty-four trees (59%, *n* = 58) were small (DBH<20 cm). Thirty-six trees (62%, *n* = 58) were classified as light wood by community monitors using a scale from one to three. Forty-one (71%, *n* = 58) of the unidentified trees were found in the primary forest on steep slopes (>45 degrees), 22% in the disturbed forest closer to the village, and 7% in the ancient tea plantations in the vicinity of the village. Twenty-two trees (37%, *n* = 58) were useful for timber. None of the unidentified trees (0%, *n* = 58) had edible fruits. Thirteen (22%, *n* = 58) did not have any known uses (Table 3 and S2 File).

**Table 3:** Characteristics of trees unidentified by community monitors compared to total number of trees in montane forests in Yunnan, China. * Light wood was classified by community monitors on a scale from 1 to 3 (low to high wood density).

Criteria	Characteristic	Proportion of unidentified trees (*n* = 58 trees)	Proportion of all trees (*n* = 1071 trees)
Taxon	*Magnoliaceae*, *Meliaceae*, *Myristicaceae*, *Rubiaceae*, and *Rutaceae*	57%	7%
Wood density	Light wood (1 of 3)*	62%	34%
Size	Small size DBH 10–20 cm	59%	47%
Habitat	Primary forest *sensu* Corlett [68]	71%	53%
Abundance	Rare (1–3 trees in the plot network of 60 vegetation plots)	78%	15%
Usefulness to the communities *sensu* [66,67]	Useful for timber	37%	30%
Usefulness to the communities *sensu* [66,67]	Fruit trees	0%	9%
Usefulness to the communities *sensu* [66,67]	Other uses	22%	49%

Community monitors had between 2 and 4 vernacular names for 29 species (18%, *n* = 161 taxa) recognised by the botanists (S1 File). For example, *Colona floribunda* is named *pao huo sheng* when using its fibres to make fires and *fan peng shu* when using its hardy and non-perishable timber to construct outdoor kitchens. Some species also had one local name for their use and another for a conspicuous characteristic. For example, *Ficus auriculata* is named both after its edible fruit and its conspicuous ear-shaped leaves and *Engelhardtia spicata* is named both for its soft and easily cut wood and for its bark, which resembles the flab around the waist of a fat woman. *Toona ciliata*, a luxury wood, has three local names; a traditional local name, a local name adopted from the Flora of China and a local name referring to the quality of the timber. If local synonyms, as in the examples above, were used consistently (*n* > 2 times) for the same scientific species, we considered the synonyms useful in identification.

### Costs of Tree Identification

Finally, we estimated the costs of tree identifications (Table 4). The costs of the plot survey were 4.5 USD/ha for botanists and 1.3 USD/ha for community monitors. Salary and domestic travel were the main expenses in the botanist-executed plot survey. The community monitors all lived in an adjacent village. Salaries paid to community monitors were higher than the daily rates in the rubber plantations due to the strenuous work on the slopes. Botanists and community monitors both spent 7 days visiting all plots. The cost of equipment was minimal and constituted spray paint for marking the plots, a pair of clippers to collect specimens and cardboard and newspapers for the plant press. The relative amount of expenses disbursed at village level was 23% for the botanist-led survey and 89% for the community-led survey (S3 File).

**Table 4:** Costs of tree species identification in the plot network by professional botanists and community monitors in montane forest in Yunnan, China (in USD). * Two villagers acted as guides and assistants during botanists’ tree identification.

	Botanists	Community monitors
Number of plots surveyed	60	60
Area surveyed (ha)	761	761
Transport incl. domestic flight (USD)	533	0
Accommodation and food (outside field site) (USD)	72	0
Accommodation and food (in field site) (USD)	264	0
Salaries professional botanists (USD)	2000	0
Salaries community monitors (USD)	528*	870
Equipment (USD)	56	56
Courier of field forms (from village to the intermediate organisation) (USD)	n.a.	50
Total cost (USD)	3453	976
Cost/plot (USD)	58	16
Cost/ha (USD)	4.5	1.3
Salaries (proportion of total cost)	73%	89%
Logistics and equipment (proportion of total cost)	27%	11%
Expenses disbursed at village level (proportion of total cost)	23%	89%

## Discussion

Our results suggest that local experts from among the Yi people can reliably identify tree species in Yunnan’s forests without having access to identification guides and herbaria. Moreover, these local experts are able to collect large volumes of tree diversity data at relatively low cost. The fact that the local experts’ and trained botanists’ results matched cannot be a result of the pre-study training exercise because this focused only on plot establishment and tree girth measurements.

How representative are our findings? We looked at the ability of experienced Yi community members to match botanists’ identifications of tree species in one area at one time. Our results are similar to field investigations in Xishuangbanna, China, and Ecuador and Brazil [14,15,63], but contrast with results from Indonesia where plant names provided by local informants, who had their experience from timber companies, could not be equated to particular taxa [12,64]. Not all local monitors are thus able to handle this task [69]. Unfortunately, few publications on the topic describe how the community monitors were chosen (Table 1). Local knowledge may vary with age, proximity to the resource and experience [70]. In the present study, the community monitors were carefully selected by representatives of the local community, based on their interest in and experience of forest resources. Moreover, the taxonomic identifications in our study were made by the late J.F. Maxwell, who was acknowledged as the most skilled field botanist in Indochina [71]. Further studies are needed to examine the replicability of our findings among carefully selected community member experts and botanists in other areas.

The tree taxa that were not identified by the community monitors differed from the majority of trees in that they were mainly light-wood, low-density taxa of primary forest, of limited use-value to the local communities. These findings concur with previous studies which demonstrate that community member assessments may be suitable for monitoring organisms or phenomena that are meaningful to community members e.g. as source of food or income or with cultural or spiritual value [12,16,34]. If the aim is to monitor attributes that are not relevant from a local perspective then local community members’ assessments may not be suitable [72].

While studies on the local uses of plants are numerous, in-depth and well-documented studies on the principles underlying folk biological taxonomy and nomenclature in non-Western societies are still lacking. This lack of understanding of the conceptual foundations of ‘ethnoscience’ (*sensu* UNESCO, [73]) as practised by non-Western people may partly explain the few attempts to bring indigenous and local knowledge systems into the science-policy interface [56, 74]. Linguistic and cultural barriers continue to hamper such efforts [34,40].

Attempts have been made to elaborate standards for monitoring biodiversity in relation to REDD+ [21,23,75]. Yet few REDD+ programs include community-level monitoring of biodiversity [47].

Accuracy is essential when forest carbon stocks are measured in order to track emissions from deforestation. Most forest carbon stock monitoring in REDD+ programs is undertaken by national consultants and foreign experts using remote sensing [76]. There may, however, be markedly divergent estimates of forest carbon density when measured from ground plots and satellites [77]. Variations in biodiversity can matter greatly when determining carbon stocks but neither wood density nor species assemblages can be mapped from space. Our findings suggest that community monitors may be important partners not only in terms of measuring stem diameters but also identifying tree species (at least to genus level), thus enabling stems to be matched to wood density information. Community monitors’ tree identifications can thus complement, and add value to, remote sensing.

In REDD+ pilot schemes where community members have been involved in monitoring forest biomass, their role has been largely limited to data collection [47,50,52,53,69,76]. In every case, an intermediate organisation has helped establish the plot network and interpret the biomass data. Since we found a high degree of one-to-one correspondence between the vernacular names and the botanists’ named taxa, an intermediate organisation could also translate the local experts’ tree data into Latin names, thereby connecting the vernacular names to the current scientific knowledge of each taxon.

A key debate surrounding the development of REDD+ relates to costs [51,76,78]. Although opportunity costs are generally considered the largest cost component of REDD+, monitoring may also form a significant component of the total project costs [78]. Our results suggest that sustaining a network of field plots is 70% cheaper (1.3 USD/ha/year) when tree diversity data are collected by community experts instead of botanists (4.5 USD/ha/year). Moreover, our findings suggest that community monitoring of canopy trees in vegetation plots resulted in 89% of all expenditure being disbursed at community level as opposed to 23% when the monitoring was led by professional scientists.

Monitoring costs at six Peruvian REDD+ sites ranged from 0.2–4.0 USD/ha/year [78]. The most important factors determining costs per hectare are: i) the size of area to be monitored [50,69,76], ii) the desired level of accuracy [78], and iii) the salary and time taken ([50], this study). The monitoring cost per ha decreases as the size of the forest area increases. The start-up costs of community monitoring may be high but, with time, the community monitors’ skills improve and community monitoring becomes a cost-effective alternative to professional forest monitoring [50]. In addition, past works suggest that community involvement in monitoring enhances feelings of ownership and improves governance while building local capacity [79–80].

There are challenges to using tree data from permanent vegetation plots to provide input to forest management. Permanent plots may be treated differently from the rest of the forest. Over time, the tree composition in the plots may therefore no longer be representative of the forest area. Moreover, if pressures on the forest are high, the frequency of data collection may not match the speed with which the forest is undergoing transformation. Monitoring of tree diversity in REDD+ programs therefore cannot stand alone. Monitoring the status of threatened species, potential threats and changes in the use of the forest and its resources, may also be necessary [81]. Participatory REDD+ programs will require complementary participatory biodiversity monitoring tools that can quickly provide reliable information with which to guide action, at a low cost. One such approach is that of focus group discussions with knowledgeable local community members on the status of particular natural resources and species of significance due to their role, value or conservation status [82].

**Fig 1:**
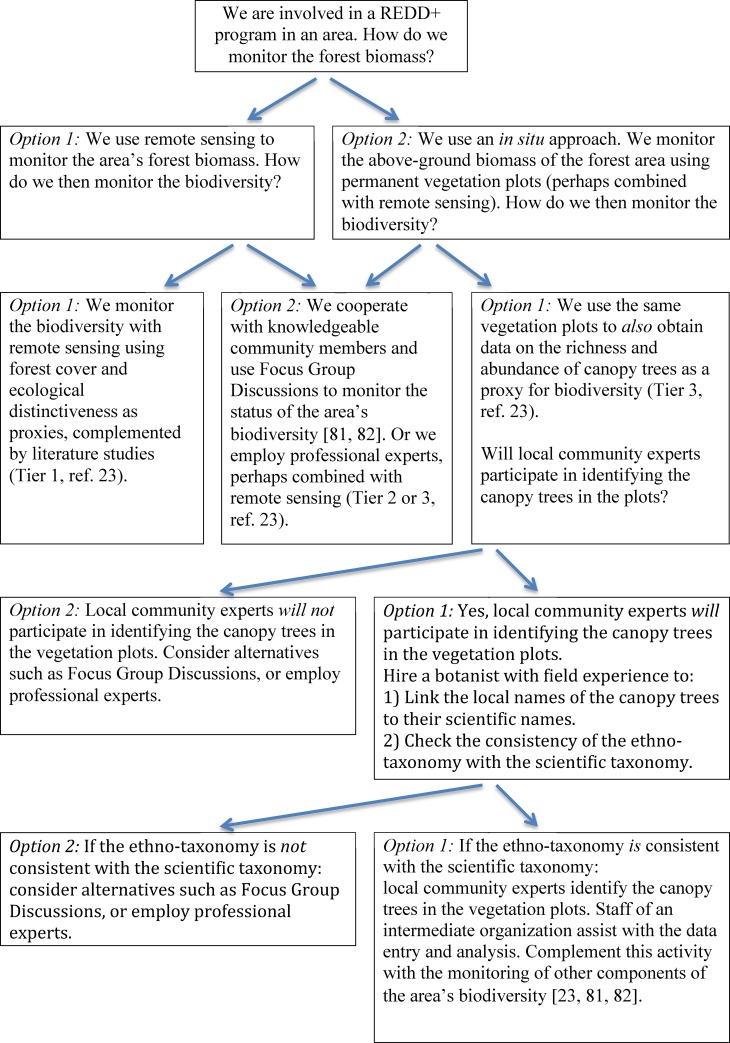
Decision tree to guide practitioners in choosing methods for biomass and biodiversity monitoring in REDD+ programs. The arrows indicate the flow of the decisions. REDD+ programs using permanent forest vegetation plots as part of their monitoring of the above-ground biomass of a forest area can take advantage of data from the same plots for monitoring the richness and abundance of canopy trees.

There was no conflict over the forest and its resources in this study. If community-based biodiversity monitoring is to become a key element in the monitoring of participatory REDD+ programs, periodic triangulation of the monitoring results will be required, although this is no different from any well-designed natural resource management initiative, whether the monitoring is implemented by communities, the government or the private sector [69]. To help practitioners choose suitable approaches for biomass and biodiversity monitoring in REDD+ programs, we have developed a decision tree (Fig 1).

There are major international efforts underway by botanists to inventory tropical forest trees [32,83,84]. Local involvement is also relevant in this context. Initial reliance on local nomenclature for documenting tree richness should be augmented with the positive identification of voucher specimens [12,34,64]. It is an arduous task to collect specimens due to the infrequent flowering of tropical plants. A long-term collection of fertile material, when available (month by month), would benefit hugely from the involvement of local experts [33,36,85].

In conclusion, we have shown that if community members with significant knowledge of forest trees are already assessing the biomass in a network of vegetation plots as part of Participatory Measurement, Reporting and Verification for REDD+ programs [48,76], then minimal further effort is required for them to collect data on the diversity of trees in the same plots. Such an approach could generate large volumes of high-quality tree diversity data at a relatively low cost.

## Supporting Information

S1 File List of species, author, local names, number of trees in plot network, wood density, and usefulness as timber, fruit, or other uses. Wood density was classified by communities on a scale from 1 to 3 (low to high wood density). Information on uses is based on Flora of Yunnan (1977–2006) and Flora of China (2014).
(DOCX)

S2 File Characteristics of the 58 trees that remained unidentified by community monitors. Information on uses is based on Flora of Yunnan (1977–2006) and Flora of China (2014). * Wood density was classified by communities on a scale from 1 to 3 (low to high wood density).
(DOCX)

S3 File Details of the costs of botanist and community-collected tree data. (DOCX)
