# Supplementary material for: Can Community Members Identify Tropical Tree Species for REDD+ Carbon and Biodiversity Measurements?
Source: PLoS One. 2016 Nov 4;11(11):e0152061. doi: 10.1371/journal.pone.0152061 (PMC5096847; doi:10.1371/journal.pone.0152061)
Supplement: S1 File — Wood density was classified by communities on a scale from 1 to 3 (low to high wood density). Information on uses is based on Flora of Yunnan (1977–2006) and Flora of China (2014). (DOCX) [file pone.0152061.s001.docx]

**Supporting file 1. List of species, local names, number of trees in plot network, wood density, and usefulness as timber, fruit, and other uses. * Wood density was classified by communities on a scale from 1 to 3 (low to high wood density). Information on uses is based on Flora of Yunnan (1977–2006) and Flora of China (2014).**

| **Family** | **Species** | **Author** | **Chinese phonetic alphabet** | **No. trees** | **Wood density*** | **Used as Timber** | **Edible fruits** | **Other uses** |
| --- | --- | --- | --- | --- | --- | --- | --- | --- |
| Aceraceae/槭树科 | Acer laurinum | Hasskarl | NA | 1 | 2 | no | no | no |
| Actinidiaceae/猕猴桃科 | Saurauia yunnanensis | C. F. Liang & Y. S. Wang | lao shui niu pao zhang hua | 3 | 1 | no | no | no |
| Alangiaceae/八角枫科 | Alangium chinense | (Loureiro) Harms | jia huang sang | 6 | 1 | yes | no | yes |
| Alangiaceae/八角枫科 | Alangium kurzii | Craib | NA | 1 | 1 | no | no | yes |
| Anacardiaceae/漆树科 | Choerospondias axillaris | (Roxburgh) B. L. Burtt & A. W. Hill | wu zhi yan guo shu | 12 | 2 | no | yes | yes |
| Anacardiaceae/漆树科 | Rhus chinensis | Miller | ye suan mao/yang suan mu | 5 | 1 | no | yes | yes |
| Anacardiaceae/漆树科 | Semecarpus reticulatus | Lecomte | da hei qi lai shu | 3 | 1 | no | no | no |
| Anacardiaceae/漆树科 | Spondias pinnata | (Linnaeus f.) Kurz | ga li luo | 2 | 1 | no | yes | yes |
| Anacardiaceae/漆树科 | Toxicodendron vernicifluum | (Stokes) F. A. Barkley | qi lai shu | 1 | 2 | yes | no | yes |
| Apocynaceae/夹竹桃科 | Alstonia rostrata | C. E. C. Fischer | NA | 1 | 3 | yes | no | yes |
| Apocynaceae/夹竹桃科 | Alstonia scholaris | (Linnaeus) R. Brown | ai tuo tuo shu/bai jiang shu | 4 | 2 | no | no | no |
| Apocynaceae/夹竹桃科 | Wrightia coccinea | (Loddiges) Sims | xiang ya mu shu | 1 | 1 | no | no | no |
| Aquifoliaceae/冬青科 | Ilex sp. | NA | hou pi shu | 4 | 1 | no | no | yes |
| Araliaceae/五加科 | Heteropanax fragrans | (Roxburgh ex Candolle) Seemann | lao xiang gan zhe shu | 2 | 1 | no | no | no |
| Araliaceae/五加科 | Macropanax undulatus | (Wallich ex G. Don) Seemann | xi ma sang guang | 4 | 1 | no | no | no |
| Araliaceae/五加科 | Schefflera sp. | NA | ma sang guang | 1 | 1 | no | no | yes |
| Araliaceae/五加科 | Trevesia palmata | (Roxburgh ex Lindley) Visiani | da ma sang guang | 4 | 1 | no | no | yes |
| Arecaceae/棕榈科 | Caryota maxima | Blume ex Martius | tie zong | 1 | 1 | no | no | yes |
| Arecaceae/棕榈科 | Caryota obtusa | Griffith | dong zong | 2 | 1 | no | no | yes |
| Asteraceae/菊科 | Vernonia volkameriifolia | Candolle | NA | 1 | 1 | yes | no | yes |
| Betulaceae/桦木科 | Betula alnoides | Buchanan-Hamilton ex D. Don | hua li mu | 16 | 3 | yes | no | yes |
| Bignoniaceae/紫葳科 | Markhamia stipulata | (Wallich) Seemann ex K. Schumann | da ku hua shu | 14 | 2 | yes | no | no |
| Bignoniaceae/紫葳科 | Mayodendron igneum | (Kurz) Kurz | xi ku hua | 1 | 2 | yes | no | no |
| Bignoniaceae/紫葳科 | Stereospermum colais | (Buchanan-Hamilton ex Dillwyn) Mabberley | deng dao shu/jia chun shu | 10 | 3 | no | no | yes |
| Bombacaceae/木棉科 | Bombax ceiba | Linnaeus | pan zhi hua | 1 | 1 | no | no | no |
| Boraginaceae/紫草科 | Cordia furcans | I. M. Johnston | po bu mu | 1 | 2 | yes | yes | yes |
| Burseraceae/橄榄科 | Canarium album | (Loureiro) Raeuschel | qing guo | 1 | 2 | yes | yes | yes |
| Burseraceae/橄榄科 | Canarium pimela | K. D. Koenig | da hei ye lan guo | 2 | 3 | no | no | no |
| Burseraceae/橄榄科 | Canarium strictum | Roxburgh | yan lan guo/qing guo | 2 | 1 | yes | yes | yes |
| Clusiaceae/藤黄科 | Cratoxylum cochinchinense | (Loureiro) Blume | huang ci shu | 33 | 2 | no | yes | yes |
| Ebenaceae/柿树科 | Diospyros kaki var. silvestris | Makino | mao shi zi/shi hua shu/ye shi hua/ye shi | 6 | 2 | no | no | no |
| Ebenaceae/柿树科 | Diospyros kerrii | Craib | xi shi hua | 1 | 2 | no | no | yes |
| Elaeocarpaceae/杜英科 | Elaeocarpus angustifolius | Blume | xiao hei guo shu | 2 | 2 | no | no | no |
| Elaeocarpaceae/杜英科 | Elaeocarpus austroyunnanensis | Hu | shan tao shu | 2 | 1 | no | no | no |
| Elaeocarpaceae/杜英科 | Elaeocarpus sikkimensis | Masters | shan tao shu | 5 | 2 | no | no | no |
| Elaeocarpaceae/杜英科 | Elaeocarpus sp. | NA | shan tao shu | 3 | 1 | no | no | no |
| Euphorbiaceae/大戟科 | Aporosa yunnanensis | (Pax & K. Hoffmann) F. P. Metcalf | zha po du zi guo/zha yao guo/zha yao shu | 17 | 2 | yes | yes | no |
| Euphorbiaceae/大戟科 | Baccaurea ramiflora | Loureiro | san ya guo/man hai guo | 6 | 1 | yes | no | no |
| Euphorbiaceae/大戟科 | Balakata baccata | (Roxburgh) Esser | aka shu/guan cai shu | 5 | 1 | yes | no | yes |
| Euphorbiaceae/大戟科 | Bischofia javanica | Blume | suan tai shu | 6 | 1 | no | no | no |
| Euphorbiaceae/大戟科 | Glochidion lanceolarium | (Roxburgh) Voigt | li sheng ye/zhai ye shu | 12 | 2 | no | no | no |
| Euphorbiaceae/大戟科 | Macaranga indica | Wight | da ba ba ye/bai ye shu | 14 | 1 | no | no | no |
| Euphorbiaceae/大戟科 | Mallotus paniculatus | (Lamarck) Muller Argoviensis | bai ye shu | 3 | 1 | yes | no | yes |
| Euphorbiaceae/大戟科 | Mallotus philippensis | (Lamarck) Muller Argoviensis | ye hong dou | 19 | 1 | no | no | no |
| Euphorbiaceae/大戟科 | Mallotus tetracoccus | (Roxburgh) Kurz | bai ye shu | 2 | 1 | no | no | no |
| Euphorbiaceae/大戟科 | Ostodes katharinae | Pax | qing ma ye shu/tu na ye | 8 | 1 | no | no | yes |
| Euphorbiaceae/大戟科 | Phyllanthus emblica | Linnaeus | gan lan shu | 10 | 3 | no | no | yes |
| Euphorbiaceae/大戟科 | Triadica cochinchinensis | Loureiro | NA | 1 | 1 | yes | no | yes |
| Fabaceae/豆科 | Acrocarpus fraxinifolius | Arnott | diao gu lu ye | 1 | 2 | yes | no | yes |
| Fabaceae/豆科 | Albizia chinensis | (Osbeck) Merrill | hei ye hao/ye hei xin shu | 16 | 3 | yes | no | yes |
| Fabaceae/豆科 | Albizia odoratissima | (Linnaeus f.) Bentham | bai ye hao | 6 | 2 | no | no | yes |
| Fabaceae/豆科 | Archidendron clypearia | (Jack) I. C. Nielsen | si ji shu | 1 | 1 | no | no | yes |
| Fabaceae/豆科 | Bauhinia acuminata | Linnaeus | bai hua shu | 20 | 1 | yes | no | no |
| Fabaceae/豆科 | Dalbergia cultrata | Graham ex Bentham | ye suan jiao | 1 | 3 | no | no | no |
| Fabaceae/豆科 | Erythrina stricta | Roxburgh | shan ci tong | 3 | 1 | no | no | no |
| Fabaceae/豆科 | Millettia leptobotrya | Dunn | gou gu tou shu | 25 | 2 | yes | no | yes |
| Fagaceae/壳斗科 | Castanopsis calathiformis | (Skan) Rehder & E. H. Wilson | pao shu li | 3 | 1 | no | no | no |
| Fagaceae/壳斗科 | Castanopsis carlesii var. spinulosa | W. C. Cheng & C. S. Chao | zhu liu dou | 14 | 2 | no | no | no |
| Fagaceae/壳斗科 | Castanopsis ceratacantha | Rehder & E. H. Wilson | chang mao zhu liu shu/yu shui man deng shu/zhu liu shu/mao zhu liu shu | 18 | 2 | no | no | no |
| Fagaceae/壳斗科 | Castanopsis clarkei | King ex J. D. Hooker | chang mao zhu liu shu/yu shui man deng shu/zhu liu shu | 6 | 2 | no | yes | yes |
| Fagaceae/壳斗科 | Castanopsis hystrix | J. D. Hooker & Thomson ex A. de Candolle | zhu liu shu/mao zhu liu shu | 2 | 3 | no | no | yes |
| Fagaceae/壳斗科 | Castanopsis mekongensis | A. Camus | da li shu/man deng shu | 138 | 3 | no | no | yes |
| Fagaceae/壳斗科 | Castanopsis tribuloides | (Smith) A. de Candolle | zhu liu shu | 1 | 2 | no | no | no |
| Fagaceae/壳斗科 | Lithocarpus fordianus | (Hemsley) Chun | NA | 1 | 2 | yes | no | yes |
| Fagaceae/壳斗科 | Lithocarpus grandifolius | (D. Don) S. N. Biswas | liu zhi shu | 23 | 3 | no | no | no |
| Fagaceae/壳斗科 | Lithocarpus pseudoreinwardtii | A. Camus | xi liu zhi shu | 3 | 2 | yes | yes | yes |
| Flacourtiaceae/大风子科 | Flacourtia indica | (N. L. Burman) Merrill | gan tian guo | 1 | 3 | no | no | no |
| Icacinaceae/茶茱萸科 | Gomphandra tetrandra | (Wallich) Sleumer | hong pi shu | 11 | 1 | yes | no | yes |
| Juglandaceae/胡桃科 | Engelhardia serrata var. cambodica | W. E. Manning | bai deng dao/pang po niang shu | 6 | 1 | yes | no | yes |
| Juglandaceae/胡桃科 | Engelhardia spicata | Leschenault ex Blume | bai deng dao/pang po niang shu | 22 | 1 | yes | no | no |
| Lauraceae/樟科 | Actinodaphne henryi | Gamble | cheng zi shu | 15 | 2 | no | no | no |
| Lauraceae/樟科 | Alseodaphne andersonii | (King ex J. D. Hooker) Kostermans | hong du zhong shu/da mu jiang zi | 3 | 2 | no | no | no |
| Lauraceae/樟科 | Alseodaphne petiolaris | (Meisner) J. D. Hooker | da du zhong shu | 7 | 2 | no | no | no |
| Lauraceae/樟科 | Cinnamomum javanicum | Blume | ye gui pi/gui pi | 2 | 2 | no | no | no |
| Lauraceae/樟科 | Cinnamomum tenuipile | Kostermans | xiang zhang mu | 10 | 2 | yes | yes | yes |
| Lauraceae/樟科 | Litsea cubeba | (Loureiro) Persoon | tai wu shu | 3 | 2 | yes | no | yes |
| Lauraceae/樟科 | Litsea glutinosa | (Loureiro) C. B. Robinson | da du zhong shu | 1 | 2 | no | no | no |
| Lauraceae/樟科 | Litsea lancifolia | (Roxburgh ex Nees) Bentham & J. D. Hooker ex Fernandez-Villar | ping tai shu | 11 | 2 | yes | no | yes |
| Lauraceae/樟科 | Litsea monopetala | (Roxburgh) Persoon | mao bai ye shu/da mu jiang zi/xiang jiang mu | 10 | 2 | no | no | no |
| Lauraceae/樟科 | Machilus tenuipilis | H. W. Li | NA | 1 | 1 | yes | no | no |
| Lauraceae/樟科 | Phoebe lanceolata | (Nees) Nees | chui ye zi shu | 2 | 2 | yes | no | no |
| Lauraceae/樟科 | Phoebe puwenensis | W. C. Cheng | huang xin nan | 31 | 2 | no | no | no |
| Lauraceae/樟科 | Phoebe sp. | NA | chui ye zi shu | 1 | 2 | no | no | no |
| Lythraceae/千屈菜科 | Duabanga grandiflora | (Roxburgh ex Candolle) Walpers | ma luo ying | 2 | 2 | no | no | yes |
| Magnoliaceae/木兰科 | Lirianthe henryi | (Dunn) N. H. Xia & C. Y. Wu | NA | 3 | 1 | yes | no | no |
| Magnoliaceae/木兰科 | Michelia baillonii | (Pierre) Finet & Gagnepain | shan gui hua/gui hua shu | 6 | 2 | yes | no | yes |
| Magnoliaceae/木兰科 | Michelia hypolampra | Dandy | NA | 3 | 2 | no | no | yes |
| Malvaceae/锦葵科 | Kydia calycina | Roxburgh | shui lan pi | 16 | 1 | yes | no | yes |
| Meliaceae/楝科 | Aphanamixis polystachya | (Wallich) R. Parker | NA | 5 | 1 | yes | no | yes |
| Meliaceae/楝科 | Chukrasia tabularis | A. Jussieu | NA | 2 | 1 | yes | no | no |
| Meliaceae/楝科 | Dysoxylum gotadhora | (Buchanan-Hamilton) Mabberley | NA | 3 | 1 | yes | no | yes |
| Meliaceae/楝科 | Melia azedarach | Linnaeus | ku lian guo | 8 | 2 | no | no | no |
| Meliaceae/楝科 | NA | NA | pao chun shu | 8 | 1 | yes | no | yes |
| Meliaceae/楝科 | Toona ciliata | M. Roemer | chun shu/ge da chun/hong chun | 10 | 2 | no | no | no |
| Moraceae/桑科 | Artocarpus lakoocha | Roxburgh | hou zi ying dai guo | 10 | 1 | no | yes | no |
| Moraceae/桑科 | Ficus auriculata | Loureiro | da mao guo shu/xiang er duo guo shu | 8 | 1 | no | no | no |
| Moraceae/桑科 | Ficus cyrtophylla | (Wallich ex Miquel) Miquel | cao ye shu/qing shu | 12 | 1 | no | no | no |
| Moraceae/桑科 | Ficus fistulosa | Reinwardt ex Blume | NA | 2 | 1 | no | no | no |
| Moraceae/桑科 | Ficus fulva | Reinwardt ex Blume | da mao guo shu | 1 | 2 | no | no | no |
| Moraceae/桑科 | Ficus hispida | Linnaeus f. | huo tong shu | 1 | 1 | no | no | no |
| Moraceae/桑科 | Ficus semicordata | Buchanan-Hamilton ex Smith | ji su zi guo/ji shi guo shu | 2 | 1 | no | no | no |
| Moraceae/桑科 | Ficus sp. | NA | huo tong shu | 3 | 1 | no | no | no |
| Moraceae/桑科 | Ficus tinctoria subsp. gibbosa | (Blume) Corner | cao ye shu/qing shu | 3 | 1 | no | no | no |
| Moraceae/桑科 | Macropanax chienii | G.Hoo | ji zhua ye | 1 | 1 | no | no | yes |
| Moraceae/桑科 | Morus macroura | Miquel | huang sang tiao | 2 | 2 | no | no | no |
| Myristicaceae/肉豆蔻科 | Horsfieldia kingii | (J. D. Hooker) Warburg | NA | 3 | 1 | no | no | no |
| Myristicaceae/肉豆蔻科 | Horsfieldia prainii | (King) Warburg | NA | 1 | 1 | no | no | yes |
| Myristicaceae/肉豆蔻科 | Knema globularia | (Lamarck) Warburg | niu wang zi shu | 3 | 1 | no | no | yes |
| Myristicaceae/肉豆蔻科 | Knema tenuinervia | W. J. de Wilde | zeng guang guo shu | 9 | 1 | no | no | yes |
| Myristicaceae/肉豆蔻科 | Myristica yunnanensis | Y. H. Li | NA | 2 | 1 | no | no | no |
| Myrsinaceae/紫金牛科 | Maesa ramentacea | (Roxburgh) A. de Candolle | NA | 1 | 1 | no | yes | yes |
| Myrtaceae/桃金娘科 | Syzygium nervosum | Candolle | yang shi guo shu | 7 | 1 | no | no | no |
| Myrtaceae/桃金娘科 | Syzygium sp1. | NA | yu shui guo shu | 3 | 2 | no | no | no |
| Oleaceae/木犀科 | Fraxinus chinensis | Roxburgh | NA | 2 | 1 | no | no | yes |
| Oxalidaceae/酢浆草科 | Averrhoa carambola | Linnaeus | wu ya guo shu | 1 | 2 | no | no | yes |
| Proteaceae/山龙眼科 | Heliciopsis henryi | (Diels) W. T. Wang | NA | 1 | 2 | no | no | no |
| Rhamnaceae/鼠李科 | Ziziphus rugosa | Lamarck | NA | 1 | 3 | no | no | no |
| Rhizophoraceae/红树科 | Carallia brachiata | (Loureiro) Merrill | mi you guo shu | 3 | 1 | no | yes | yes |
| Rosaceae/蔷薇科 | Cerasus serrula | (Franchet) T. T. Yu & C. L. Li | ying tao | 1 | 2 | no | no | no |
| Rosaceae/蔷薇科 | Docynia delavayi | (Franchet) C. K. Schneider | duo yi | 1 | 2 | no | yes | no |
| Rosaceae/蔷薇科 | Laurocerasus undulata | (Buchanan-Hamilton ex D. Don) M. Roemer | NA | 1 | 2 | no | no | no |
| Rosaceae/蔷薇科 | Pygeum henryi | Dunn | NA | 1 | 2 | no | no | no |
| Rubiaceae/茜草科 | Tarennoidea wallichii | (J. D. Hooker) Tirvengadum & Sastre | NA | 8 | 3 | yes | no | no |
| Rubiaceae/茜草科 | Wendlandia tinctoria subsp. barbata | Cowan | xi hong mao shu | 14 | 2 | no | no | no |
| Rutaceae/芸香科 | Micromelum integerrimum | (Buchanan-Hamilton ex Candolle) Wight & Arnott ex M. Roemer | NA | 3 | 1 | no | no | yes |
| Rutaceae/芸香科 | Tetradium austrosinense | (Handel-Mazzetti) T. G. Hartley | ye ma qing/pao chun shu | 6 | 1 | no | no | no |
| Rutaceae/芸香科 | Tetradium trichotomum | Loureiro | ye la zi shu | 3 | 1 | no | no | yes |
| Sabiaceae/清风藤科 | Meliosma arnottiana | (Wight) Walpers | pao chun shu | 14 | 1 | no | no | no |
| Santalaceae/檀香科 | Scleropyrum wallichianum | (Wight & Arnott) Arnott | shan he tao guo | 1 | 2 | no | no | yes |
| Sapindaceae/无患子科 | Arytera littoralis | Blume | mai nu nan | 1 | 1 | yes | no | yes |
| Sapindaceae/无患子科 | Nephelium chryseum | Blume | mao li zhi shu/yang guo shu/qian guo shu | 5 | 2 | no | no | yes |
| Sapindaceae/无患子科 | Pometia pinnata | J. R. Forster & G. Forster | hong mei ga | 3 | 3 | no | no | no |
| Sapindaceae/无患子科 | Sapindus rarak | Candolle | pi shao zi | 10 | 2 | no | no | yes |
| Sapotaceae/山榄科 | Pouteria grandifolia | (Wallich) Baehni | lan cang guo | 1 | 1 | no | yes | no |
| Sapotaceae/山榄科 | Sarcosperma kachinense | (King & Prain) Exell | NA | 1 | 1 | no | no | yes |
| Sapotaceae/山榄科 | Sarcosperma laurinum | (Bentham) J. D. Hooker | ji xin guo shu | 10 | 1 | yes | no | no |
| Simaroubaceae/苦木科 | Picrasma chinensis | P. Y. Chen | pao chun shu | 1 | 1 | no | no | no |
| Staphyleaceae/省沽油科 | Turpinia pomifera | (Roxburgh) Candolle | ye duo yi | 6 | 2 | no | no | no |
| Theaceae/山茶科 | Eurya groffii | Merrill | da shu sui mi | 1 | 2 | no | no | no |
| Theaceae/山茶科 | Schima argentea | E. Pritzel | hong mao shu | 5 | 3 | no | no | no |
| Theaceae/山茶科 | Schima wallichii | (Candolle) Korthals | hong mao shu | 109 | 3 | no | no | no |
| Tiliaceae/椴树科 | Colona floribunda | (Wallich ex Kurz) Craib | pao huo sheng/fan peng shu | 3 | 1 | no | no | yes |
| Ulmaceae/榆科 | Celtis tetrandra | Roxburgh | zi geng shu | 1 | 2 | no | no | no |
| Ulmaceae/榆科 | Celtis timorensis | Spanoghe | mao tie chi | 4 | 2 | no | no | no |
| Ulmaceae/榆科 | Trema tomentosa | (Roxburgh) H. Hara | duan ming shu | 1 | 1 | yes | no | yes |
| Urticaceae/荨麻科 | Debregeasia orientalis | C. J. Chen | ma ye shu | 3 | 1 | no | yes | yes |
| Verbenaceae/马鞭草科 | Gmelina arborea | Roxburgh | xiang hua shu | 4 | 2 | yes | no | yes |
| Verbenaceae/马鞭草科 | Vitex negundo | Linnaeus | wu ya ye | 2 | 1 | no | no | yes |
| Verbenaceae/马鞭草科 | Vitex peduncularis | Wallich ex Schauer | NA | 1 | 1 | no | no | yes |
| Verbenaceae/马鞭草科 | Vitex quinata var. puberula | (H. J. Lam) Moldenke | wu ya ye | 6 | 1 | no | no | yes |
| Unknown | Unknown sp 1 | NA | hang di shu | 1 | 1 | no | no | no |
| Unknown | Unknown sp 2 | NA | lao wang guo | 1 | 1 | no | no | no |
| Unknown | Unknown sp 3 | NA | niu lei ba shu | 1 | 2 | no | no | no |
| Unknown | Unknown sp 4 | NA | tong hua shu | 1 | 1 | no | no | no |
| Unknown | Unknown sp 5 | NA | NA | 1 | 1 | no | no | no |
| Unknown | Unknown sp 6 | NA | NA | 1 | 1 | no | no | no |
| Unknown | Unknown sp 7 | NA | NA | 1 | 1 | no | no | no |
| Unknown | Unknown sp 8 | NA | NA | 1 | 1 | no | no | no |
| Unknown | Unknown sp 9 | NA | NA | 1 | 1 | no | no | no |
| Unknown | Unknown sp 10 | NA | NA | 1 | 1 | no | no | no |
| Unknown | Unknown sp 11 | NA | NA | 1 | 1 | no | no | no |
| **Total (n)** | **161** |  | **111** | **1071** |  | **43** | **18** | **70** |
