# Supplementary material for: Can Community Members Identify Tropical Tree Species for REDD+ Carbon and Biodiversity Measurements?
Source: PLoS One. 2016 Nov 4;11(11):e0152061. doi: 10.1371/journal.pone.0152061 (PMC5096847; doi:10.1371/journal.pone.0152061)
Supplement: S2 File — Information on uses is based on Flora of Yunnan (1977–2006) and Flora of China (2014). * Wood density was classified by communities on a scale from 1 to 3 (low to high wood density). (DOCX) [file pone.0152061.s002.docx]

**Supporting file 2. Characteristics for the 58 trees which remained unidentified by community monitors. Information on uses is based on Flora of Yunnan (1977–2006) and Flora of China (2014). * Wood density was classified by communities on a scale from 1 to 3 (low to high wood density).**

| **Family** | **Species** | **Author** | **No. trees** | **DBH (cm)** | **Wood density*** | **Habitat** | **Used as Timber** | **Edible fruits** | **Other uses** |
| --- | --- | --- | --- | --- | --- | --- | --- | --- | --- |
| Aceraceae/槭树科 | Acer laurinum | Hasskarl | 1 | 10.7 | 2 | Primary Forest | no | no | no |
| Alangiaceae/八角枫科 | Alangium kurzii | Craib | 1 | 20.8 | 1 | Secondary | no | no | yes |
| Apocynaceae/夹竹桃科 | Alstonia rostrata | C. E. C. Fischer | 1 | 59.0 | 3 | Primary Forest | yes | no | yes |
| Asteraceae/菊科 | Vernonia volkameriifolia | Candolle | 1 | 11.1 | 1 | Primary Forest | no | no | yes |
| Euphorbiaceae/大戟科 | Triadica cochinchinensis | Loureiro | 1 | 21.3 | 1 | Secondary, tea | no | no | yes |
| Fagaceae/壳斗科 | Lithocarpus fordianus | (Hemsley) Chun | 1 | 27.7 | 2 | Primary Forest | no | no | no |
| Lauraceae/樟科 | Machilus tenuipilis | H. W. Li | 1 | 15.9 | 1 | Primary Forest | no | no | no |
| Magnoliaceae/木兰科 | Lirianthe henryi | (Dunn) N. H. Xia & C. Y. Wu | 3 | 15.1 | 1 | Primary Forest | no | no | yes |
| Magnoliaceae/木兰科 | Lirianthe henryi | (Dunn) N. H. Xia & C. Y. Wu |  | 12.1 | 1 | Primary Forest | no | no | yes |
| Magnoliaceae/木兰科 | Lirianthe henryi | (Dunn) N. H. Xia & C. Y. Wu |  | 17.9 | 1 | Primary Forest | no | no | yes |
| Magnoliaceae/木兰科 | Michelia hypolampra | Dandy | 3 | 39.9 | 2 | Secondary | yes | no | yes |
| Magnoliaceae/木兰科 | Michelia hypolampra | Dandy |  | 71.6 | 2 | Primary forest | yes | no | yes |
| Magnoliaceae/木兰科 | Michelia hypolampra | Dandy |  | 21.0 | 1 | Primary forest | yes | no | yes |
| Meliaceae/楝科 | Aphanamixis polystachya | (Wallich) R. Parker | 5 | 24.1 | 1 | Secondary | yes | no | yes |
| Meliaceae/楝科 | Aphanamixis polystachya | (Wallich) R. Parker |  | 50.5 | 2 | Primary Forest | yes | no | yes |
| Meliaceae/楝科 | Aphanamixis polystachya | (Wallich) R. Parker |  | 13.7 | 1 | Primary Forest | yes | no | yes |
| Meliaceae/楝科 | Aphanamixis polystachya | (Wallich) R. Parker |  | 17.4 | 1 | Primary Forest | yes | no | yes |
| Meliaceae/楝科 | Aphanamixis polystachya | (Wallich) R. Parker |  | 19.0 | 2 | Secondary | yes | no | yes |
| Meliaceae/楝科 | Chukrasia tabularis | A. Jussieu | 2 | 17.8 | 1 | Secondary, tea | yes | no | yes |
| Meliaceae/楝科 | Chukrasia tabularis | A. Jussieu |  | 10.1 | 1 | Secondary | yes | no | yes |
| Meliaceae/楝科 | Dysoxylum gotadhora | (Buchanan-Hamilton) Mabberley | 3 | 22.0 | 1 | Primary Forest | yes | no | no |
| Meliaceae/楝科 | Dysoxylum gotadhora | (Buchanan-Hamilton) Mabberley |  | 12.8 | 2 | Primary Forest | yes | no | no |
| Meliaceae/楝科 | Dysoxylum gotadhora | (Buchanan-Hamilton) Mabberley |  | 15.2 | 2 | Primary Forest | yes | no | no |
| Moraceae/桑科 | Ficus fistulosa | Reinwardt ex Blume | 2 | 13.3 | 1 | Primary Forest | no | no | no |
| Moraceae/桑科 | Ficus fistulosa | Reinwardt ex Blume |  | 15.3 | 1 | Primary Forest | no | no | no |
| Myristicaceae/肉豆蔻科 | Horsfieldia kingii | (J. D. Hooker) Warburg | 3 | 15.1 | 1 | Primary forest | no | no | no |
| Myristicaceae/肉豆蔻科 | Horsfieldia kingii | (J. D. Hooker) Warburg |  | 33.0 | 1 | Primary forest | no | no | no |
| Myristicaceae/肉豆蔻科 | Horsfieldia kingii | (J. D. Hooker) Warburg |  | 11.6 | 2 | Primary Forest | no | no | no |
| Myristicaceae/肉豆蔻科 | Horsfieldia prainii | (King) Warburg | 1 | 41.1 | 1 | Secondary | no | no | no |
| Myristicaceae/肉豆蔻科 | Myristica yunnanensis | Y. H. Li | 2 | 11.6 | 1 | Secondary | no | no | yes |
| Myristicaceae/肉豆蔻科 | Myristica yunnanensis | Y. H. Li |  | 22.3 | 1 | Secondary, tea | no | no | yes |
| Myrsinaceae/紫金牛科 | Maesa ramentacea | (Roxburgh) A. de Candolle | 1 | 27.5 | 1 | Primary forest | no | no | no |
| Oleaceae/木犀科 | Fraxinus chinensis | Roxburgh | 2 | 30.9 | 1 | Secondary, tea | no | no | yes |
| Oleaceae/木犀科 | Fraxinus chinensis | Roxburgh |  | 10.4 | 1 | Primary forest | no | no | yes |
| Proteaceae/山龙眼科 | Heliciopsis henryi | (Diels) W. T. Wang | 1 | 35.6 | 2 | Primary forest | no | no | no |
| Rhamnaceae/鼠李科 | Ziziphus rugosa | Lamarck | 1 | 14.7 | 3 | Secondary | no | no | no |
| Rosaceae/蔷薇科 | Laurocerasus undulata | (Buchanan-Hamilton ex D. Don) M. Roemer | 1 | 20.9 | 2 | Primary Forest | no | no | no |
| Rosaceae/蔷薇科 | Pygeum henryi | Dunn | 1 | 18.1 | 2 | Secondary | no | no | no |
| Rubiaceae/茜草科 | Tarennoidea wallichii | (J. D. Hooker) Tirvengadum & Sastre | 8 | 43.9 | 3 | Primary Forest | yes | no | no |
| Rubiaceae/茜草科 | Tarennoidea wallichii | (J. D. Hooker) Tirvengadum & Sastre |  | 15.5 | 3 | Primary Forest | yes | no | no |
| Rubiaceae/茜草科 | Tarennoidea wallichii | (J. D. Hooker) Tirvengadum & Sastre |  | 22.9 | 3 | Primary Forest | yes | no | no |
| Rubiaceae/茜草科 | Tarennoidea wallichii | (J. D. Hooker) Tirvengadum & Sastre |  | 12.0 | 3 | Primary Forest | yes | no | no |
| Rubiaceae/茜草科 | Tarennoidea wallichii | (J. D. Hooker) Tirvengadum & Sastre |  | 10.0 | 3 | Primary Forest | yes | no | no |
| Rubiaceae/茜草科 | Tarennoidea wallichii | (J. D. Hooker) Tirvengadum & Sastre |  | 20.4 | 3 | Primary Forest | yes | no | no |
| Rubiaceae/茜草科 | Tarennoidea wallichii | (J. D. Hooker) Tirvengadum & Sastre |  | 36.5 | 3 | Primary Forest | yes | no | no |
| Rubiaceae/茜草科 | Tarennoidea wallichii | (J. D. Hooker) Tirvengadum & Sastre |  | 43.4 | 3 | Primary Forest | yes | no | no |
| Rutaceae/芸香科 | Micromelum integerrimum | (Buchanan-Hamilton ex Candolle) Wight & Arnott ex M. Roemer | 3 | 18.4 | 1 | Secondary | no | no | yes |
| Rutaceae/芸香科 | Micromelum integerrimum | (Buchanan-Hamilton ex Candolle) Wight & Arnott ex M. Roemer |  | 11.5 | 1 | Secondary | no | no | yes |
| Rutaceae/芸香科 | Micromelum integerrimum | (Buchanan-Hamilton ex Candolle) Wight & Arnott ex M. Roemer |  | 13.6 | 1 | Secondary | no | no | yes |
| Sapotaceae/山榄科 | Sarcosperma kachinense | (King & Prain) Exell | 1 | 23.0 | 1 | Primary Forest | no | no | yes |
| Verbenaceae/马鞭草科 | Vitex peduncularis | Wallich ex Schauer | 1 | 19.5 | 1 | Primary Forest | no | no | yes |
| Unknown | Unknown265 | NA | 1 | 10.0 | 1 | Primary Forest | no | no | no |
| Unknown | Unknown360 | NA | 1 | 46.8 | 1 | Primary Forest | no | no | no |
| Unknown | Unknown465 | NA | 1 | 16.7 | 1 | Secondary | no | no | no |
| Unknown | Unknown701 | NA | 1 | 31.7 | 1 | Primary Forest | no | no | no |
| Unknown | Unknown767 | NA | 1 | 17.3 | 1 | Primary forest | no | no | no |
| Unknown | Unknown965 | NA | 1 | 14.8 | 1 | Primary forest | no | no | no |
| Unknown | Unknown992 | NA | 1 | 15.3 | 1 | Primary Forest | no | no | no |
