# Supplementary material for: Can Community Members Identify Tropical Tree Species for REDD+ Carbon and Biodiversity Measurements?
Source: PLoS One. 2016 Nov 4;11(11):e0152061. doi: 10.1371/journal.pone.0152061 (PMC5096847; doi:10.1371/journal.pone.0152061)
Supplement: S3 File — (DOCX) [file pone.0152061.s003.docx]

**Supporting file 3. Details on the costs of botanist and community collected tree data.**

Calculations of the costs of botanist and community tree identifications were prepared based on actual costs incurred in the field. All values are in USD at exchange rates as of January 2015: Rmb 1 = USD 0.15 (January 2015).

**Tree identifications by botanists (2012).**

Transport including domestic flight: Airfare Kunming-Jinghong return : USD 333. Taxi Jinghong-Manlin return USD 200. In total USD 533.

Accommodation and food outside field site: Guest house and food for overnight stay in Jinghong on way to field site and visiting the herbarium at Xishuangbanna Botanical Garden on return from field site amounted to USD 72.

Accommodation and food within field site (Manlin village): The botanist stayed in a private home for USD 10 per day for 11 days. In total USD 110. Food was prepared by villagers in Manlin for USD 14 per day for 11 days. In total USD 154. Altogether accommodation and food within the field site amounted to USD 264.

Salary botanist: An honorarium was paid to the chief botanist for 11 days of fieldwork and 4 days of herbaria work. The assistant botanist was a PhD fellow and his salary and expenses was paid by his stipend. We believe that one botanist possibly assisted by a student is a realistic set-up in future REDD+ monitoring of tree diversity. Total cost: USD 2000.

Salary for community monitors: Two community monitors where paid USD 24 per day for 11 days as guides and field assistants during the botanist-led survey. The community monitors assisted on specimen collection and provided information on flowering and fruiting. In total USD 528.

Equipment: The plot network was established beforehand for AGB measurements. Two clippers, alhohol to preserve specimens in the field, cardboard and newspapers for the plant press USD 56.

**Tree identifications by community monitors (2013).**

Accommodation and food: No accommodation was required as community monitors were all from Manlin village situated next to the forest. The plot-survey was done by day-trips to the forest. Community members took responsibility for their own food (packed lunch).

Salary for professional botanist: the community monitors were trained in plot-based forest biomass monitoring. This included recording of DBH and vernacular species name. No further training by outsiders was needed.

Salaries for community monitors: Three villagers was paid USD 29 per day for 10 days to identify trees in the forest plots. The payment was agreed beforehand. In practice, villagers took 7 days to complete the survey. In total USD 870.

Equipment: GPS devices were borrowed. Paint, measuring tape, rope and batteries amounted to USD 56.

Retrieval of plot data from village to research institute: In the present study, the research team carried the field forms back to the research institution (KIB). We investigated the price for forwarding the field forms with courier. The data sheets could be couriered from Manlin Village to Kunming Botanical Institute for USD 50. This amount has been used in the cost calculation (Table 4).
